# Environmental Policy and Household Behaviour: Sustainability and Everyday Life

**DOI:** 10.1289/ehp.119-a364

**Published:** 2011-08-01

**Authors:** Ida Ferrara

**Affiliations:** Ida Ferrara is an Associate Professor of Economics at York University, Canada. Her work focuses on environmental issues, including waste generation and recycling. Her publication outlets include *Journal of Environmental Economics and Management*, *Environmental and Resource Economics*, *Ecological Economics*, and *Canadian Public Policy*.

*Environmental Policy and Household Behaviour* is a collection of studies on household behavior in environment-related areas originated from a multidisciplinary research program carried out by four Swedish universities between 2003 and 2008 under the financial support of the Swedish Research Council for Environment, Agricultural Sciences and Spatial Planning, and the Swedish Environmental Protection Agency.

The book’s contributions can be best appreciated within the context of recent shifts in environmental policy. Over the past decade, much of the focus of environmental policy has moved away from industrial production as the main source of pollution and from the implementation of cleaner processes toward an integrated approach that considers the life-cycle environmental impacts of goods and services involving all actors along the product chain, including consumers. Environmental policy-making bodies have also recognized the importance of a better understanding of these decision-making processes and households within a broader social science framework.

For environmental policy, then, the main contribution lies in the focus on incorporating consumption-based measures into environmental policy and management and promoting more sustainable patterns of consumption a better understanding of the role and drives of consumption. For household behavior, the main contribution lies in the focus on capturing a wider range of policy influences through the inclusion of noneconomic explanations of behavior—from those grounded on noncognitive foundations of everyday life to those embedded in the collective, cultural, and social view of both choice and action.

Overall, the book is a valuable source of information for both researchers and policy makers interested in or working on environmental issues that arise in household decisions, although it may not readily appeal to researchers engaged in theoretical or methodologically sophisticated empirical analyses. It can also serve as a supplementary text in an upper-level undergraduate course in environmental policy, particularly in interdisciplinary programs such as environmental studies.

Through its comprehensive approach to household behavior, the book provides insight into the complexity of designing environmental policies to achieve environmentally sustainable lifestyles and offers guidelines as to how such a complexity can be simplified. It begins with a discussion of the potential tension between individual freedom of choice and environmental obligations and how such a conflict is avoided through market-based policy instruments that place environmental responsibility on individuals as consumers in the marketplace. It explores how households handle the complexities and challenges of making environmentally informed decisions through compensatory measures and simplifications. To help households, the authors suggests that policies should facilitate comparison across the effects of different environmental activities. The book addresses the question of policy legitimacy, which requires that policies reflect society’s core values and attitudes, and how financial rewards and punishments may be inconsistent (and thus viewed as lacking legitimacy) with a moral willingness to do the right thing.

The book provides a thorough review of four types of determinants of behavior (attitudinal factors, habits or routines, contextual factors, and personal capabilities) within a broad framework of analysis. The discussion of the various types of determinants of behavior is most enlightening in bringing together insights about human behavior from many academic disciplines and fields and also encompassing linkages between different types of factors, particularly between attitudinal factors and contextual factors and habits. Hence, inner motivation to “act green” is not likely to result in green behavior if the perceived cost of sustainable behavior is high and/or habits of nonsustainable behavior are strong.

In a series of case studies, the book analyzes the interplay of the four types of casual factors in specific environment-related household consumption areas: waste management, eco-consumption, and transportation. The three areas are cleverly selected to stress how policy challenges differ across areas as the relevance of the different casual factors and strength of interaction between them vary across different household activities. The specific reference to Sweden and the Swedish experience is most telling in illuminating another layer of complexity in the pursuit of sustainable consumption patterns that arises when nonenvironmental policies (e.g., social housing policies) and other public initiatives (e.g., improved road construction and new parking space) are at odds with promoting environmental sustainable household behavior.

Despite the relevance of combining elements about human nature and behavior from various social sciences, the book tends to overly discount, at least implicitly, the importance of economic motives and instruments. One of the three key implications summarized in the concluding chapter does speak of the need for policy packages as a potentially effective way to respond to morality-based motivations for green behavior while providing economic incentives. Nevertheless, the theoretical part of the book tends to emphasize the political, social, and psychological dimensions of household decision making, pointing to an excessive reliance on economic instruments; and the empirical part tends to overlook the impact of market-based policies even though such policies have received much attention, especially in the transport area, for their efficiency and their financial transparency.

In sum, *Environmental Policy and Household Behaviour* makes a significant contribution to our understanding of the incentives and constraints households face when formulating judgments and making decisions. This understanding is a key requirement for the design of policies that are both effective and acceptable. The book does not offer specific policy prescriptions but suggests a framework that allows for the inclusion of a wider range of motives for environmentally responsible behavior and gives the opportunity of examining and rethinking the interaction between different types of motivation.

